# Detection of Doppler Microembolic Signals Using High Order Statistics

**DOI:** 10.1155/2016/3243290

**Published:** 2016-12-14

**Authors:** Maroun Geryes, Sebastien Ménigot, Walid Hassan, Ali Mcheick, Jamal Charara, Jean-Marc Girault

**Affiliations:** ^1^Université François Rabelais de Tours, UMR Imagerie et Cerveau Inserm U930, Tours, France; ^2^Department of Physics and Electronics, Faculty of Sciences I, Lebanese University, Beirut, Lebanon; ^3^Clarivate Analytics, Dubai, UAE

## Abstract

Robust detection of the smallest circulating cerebral microemboli is an efficient way of preventing strokes, which is second cause of mortality worldwide. Transcranial Doppler ultrasound is widely considered the most convenient system for the detection of microemboli. The most common standard detection is achieved through the Doppler energy signal and depends on an empirically set constant threshold. On the other hand, in the past few years, higher order statistics have been an extensive field of research as they represent descriptive statistics that can be used to detect signal outliers. In this study, we propose new types of microembolic detectors based on the windowed calculation of the third moment skewness and fourth moment kurtosis of the energy signal. During energy embolus-free periods the distribution of the energy is not altered and the skewness and kurtosis signals do not exhibit any peak values. In the presence of emboli, the energy distribution is distorted and the skewness and kurtosis signals exhibit peaks, corresponding to the latter emboli. Applied on real signals, the detection of microemboli through the skewness and kurtosis signals outperformed the detection through standard methods. The sensitivities and specificities reached 78% and 91% and 80% and 90% for the skewness and kurtosis detectors, respectively.

## 1. Introduction

Sudden intensity increases in the Transcranial Doppler (TCD) signal are majorly interpreted as signatures resulting from cerebral emboli. The passage of cerebral emboli through blood vessels feeding the brain could result in blockage of these vessels and consequently lead to stroke, the second cause of mortality worldwide. Embolic strokes constitute up to 14% of all strokes [1]. Therefore, embolic strokes represent a major death threat and thus the early detection of the smallest microemboli is an important issue for which robust solutions must be found. This early detection would be a basis for early stroke diagnosis and thus avoiding its occurrence. Nowadays, TCD is considered the most effective embolic stroke diagnosis system.

Although the characteristics and physical nature of embolic signals, in the TCD signal, have been well defined, the task of detecting embolic and particularly small microembolic signals still poses a tough challenge. The gold standard method of detecting the passage of emboli is the audible detection of the sudden “chirp” or “moan” produced by emboli as well as the visual detection of the time-frequency representation (spectrogram) generated on the TCD screen. A main limitation of the gold standard is the inability to audibly detect microembolic signals located at the systolic phase due to temporal and frequency masking effects in audio files.

The standard signal processing method of detecting embolic signals is based on calculating the energy from the spectrogram and applying constant thresholds to pick up the emboli which, according to Rayleigh theory [2], backscatter ultrasound energy higher than that backscattered by the surrounding blood. The major limitations in standard techniques reside in the inability of detecting small microembolic signals having lower intensities than the surrounding background blood mainly at the systolic peak.

As a purpose to detect the smallest microemboli, many research works have been carried out. We list some of the most punctual methods. Frequency filtering methods were introduced in [3, 4]. The study reported high detection sensitivity and specificity rates. Subsequently, an online automated embolic signal detection algorithm based on frequency filtering was developed in [5, 6]. The latter system showed high performances in terms of sensitivity and specificity for particular cases (postcarotid endarterectomy). However, in other conditions (atrial fibrillation) the system's sensitivity and specificity severely decreased. Moreover, the system's performance in the detection of low energy microembolic signals was arguably less efficient with much lower sensitivity and specificity. Methods based on detection of sudden changes were introduced in [7]. Nonparametric detection methods mainly the Fourier, Wigner-Ville, and wavelet approaches were compared to parametric autoregressive methods. The new parametric methods were proven to be highly performant and efficient in the detection of small microemboli. However, the methods were tested on synthetic simulated Doppler signals and never on a set of real signals. Another highly productive wavelet-based system was established in [8]. The system achieved a high combination of sensitivity and specificity. However, the system's rates decreased in the case of low energy microembolic signals. A remarkable offline detection was proposed in [9]. The system had excellent performance for emboli having high intensities relative to background blood clutter. However, it is to be noticed that the study did not take into consideration the detection of weak embolic signals. The authors in [10] introduced another highly achieving detection procedure based on the discrete wavelet transform (DWT). DWT allowed major increases in specificity and sensitivity. Nonetheless, a major deficiency of the DWT implementation was the reduced frequency resolution at low frequency scales, in which embolic signals are mostly found. In [11], the authors proposed embolic detection using the adaptive wavelet packet basis and neurofuzzy classification. The adaptive wavelet packet basis was used to make a sparse representation of Doppler ultrasound blood flow signals. The method produced highly accurate and robust performances. However when compared to other methods only the sensitivity was taken into account and the correlated specificity was not calculated. The study submitted in [12] requested the use of Fractional Fourier Transform rather than the short time Fourier transform, the standard method of detection in TCD systems. The results showed that discriminating parameters based on the Fractional Fourier Transform help easier analysis and detection of embolic signals. Despite its simplicity and acceptable results, this method was not proven reliably decent for the detection of the smallest microemboli. The method proposed in [13] achieved very high sensitivity and specificity but large detection errors occurred due to small gaseous emboli exhibiting small reflected signals.

In most articles previously introduced, the main limitation lies in the fact that the information on which the detection takes place is time-varying while the threshold used is constant. To match between the time-varying information and the threshold, two solutions can be proposed. The first is proposing a time-varying threshold as in [14, 15] that matches the time-varying trend of the decision information. Second is proposing a constant threshold that matches the decision information for which the time-varying trend is removed.

In this work, the methods we proposed of matching between a constant threshold and an energy free of its time-varying trend are based on the use of high order statistic (HOS) of windowed Doppler energy signal. We tend to prove the skewness and kurtosis as two solid means to detect microembolic signals when asymptomatic caroid artery patients are monitored with a Holter TCD.

## 2. The Offline Microembolic Detection Unit

As previously mentioned, our objective is to perceive a microemboli detector more sensitive and robust regarding most standard detectors.

In this study, the typical offline signal processing unit is decomposed into 3 units:

- Unit A, allocated for loading the wave file, 10-second signal segmentation, short time Fourier transform (STFT) calculation, and instantaneous energy calculation from the STFT
- Unit B, allocated for standard energy detection on the energy signal obtained in Unit A
- Unit C, allocated for the new energy detection techniques based on skewness and kurtosis calculation of the energy signal obtained in Unit A

### 2.1. Unit A: Doppler Signal Extraction, STFT, and Instantaneous Energy Calculation

**Figure 1:**
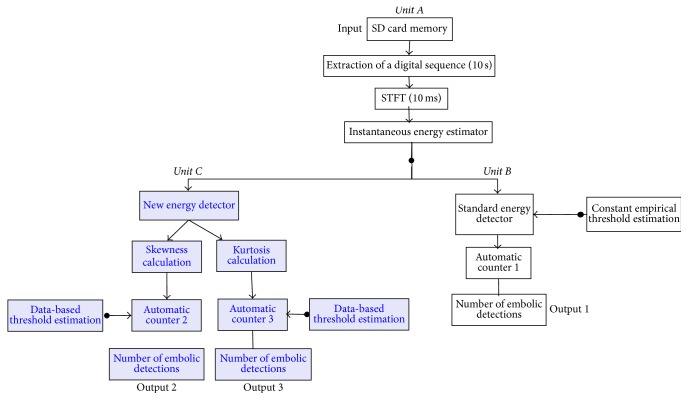
A typical embolus detection system including standard detection and our new detection procedure. Unit A includes extracting 10 s digital Doppler signal sequences from the SD card extracted from the Holter system, calculating the short time Fourier transform, and lastly calculating the instantaneous energy from STFT estimators. Unit B represents the detection achieved using standard methods while Unit C represents the new detection procedure we have developed based on skewness and kurtosis calculation.

The different systems that we want to test, depicted in Figure 1, share a common structure. From the SD card plugged out from the Holter system and plugged into the personal computer, the Doppler signal is picked up and put in memory. From this Doppler digital signal, the short time Fourier transform is calculated, first to display the spectrogram and second to estimate instantaneously the Doppler energy. Calculations of the STFT and the instantaneous energy are carried out repetitively on 10-second segments extracted from the Doppler signal.

Most commercial TCD ultrasound systems are based on the short time Fourier transform. The short time Fourier transform is an adapted form of the Fourier transform that analyzes only a small segment of the signal at a time, a technique called windowing of the signal or also Windowed Fourier Transform (WFT). Short time Fourier transform is used when the Doppler signal within the analyzing window is stationary. In reality, transforming data into the frequency domain results in loss of time information. By applying the Fourier transform of a signal, it is impossible to identify when a particular event takes place. The STFT was thus proposed to correct this deficiency. The STFT maps a signal into a two-dimensional function of time and frequency. This representation is known as the spectrogram.

The STFT frequency estimator with a sliding window can be formally written as follows:

(1) $$\begin{array}{c} S\left(\;t,f\;\right)={\left|\;\int x\left(\;\tau \;\right)w^{*}\left(\;t-\tau \;\right)\exp^{-j2\pi \mathrm{ft}}d\tau \;\right|}^{2}, \end{array}$$

where *x*(*t*) is the analyzed Doppler signal, *w*(*t*) is a sliding window, and *∗* stands for complex conjugation.

When using the STFT to process embolic signals, it is of great importance that the STFT parameters are optimized. The three processing parameters are the window size, the window type, and the overlap ratio. Despite the fact that setting the parameters significantly affects the embolus detection system based on STFT calculations, little work on the effect of the different parameters has been reported. A fundamental work was reported in [16]. The authors evaluated the effect of varying the three parameters on embolic signal temporal and frequency resolutions, time of embolic signal onset, and the power of the embolus at the frequency with maximum power relative to the average power of the background intensity. Based on [16] and after a preliminary stage of experimental optimization of the STFT parameters, the STFT in this study is performed using a 14.6-millisecond Hamming window with an overlap of 65%.

The instantaneous energy at a fixed time *t* can be obtained from STFT frequency estimators in (1) by

(2) $$\begin{array}{c} e\left(\;t\;\right)=\int S\left(\;t,f\;\right)df. \end{array}$$

Note that the energy returned by a microembolus would be greater than that returned by billions of red blood cells (RBCs), since a microembolus is often larger than RBCs. Hence, the backscattered energy would function as a solid indicator from which the presence of embolic and microembolic signatures could be detected. This justifies why most detectors are chosen to be mainly based on energy criteria.

### 2.2. Unit B: Standard Microembolic Detection

**Figure 2:**
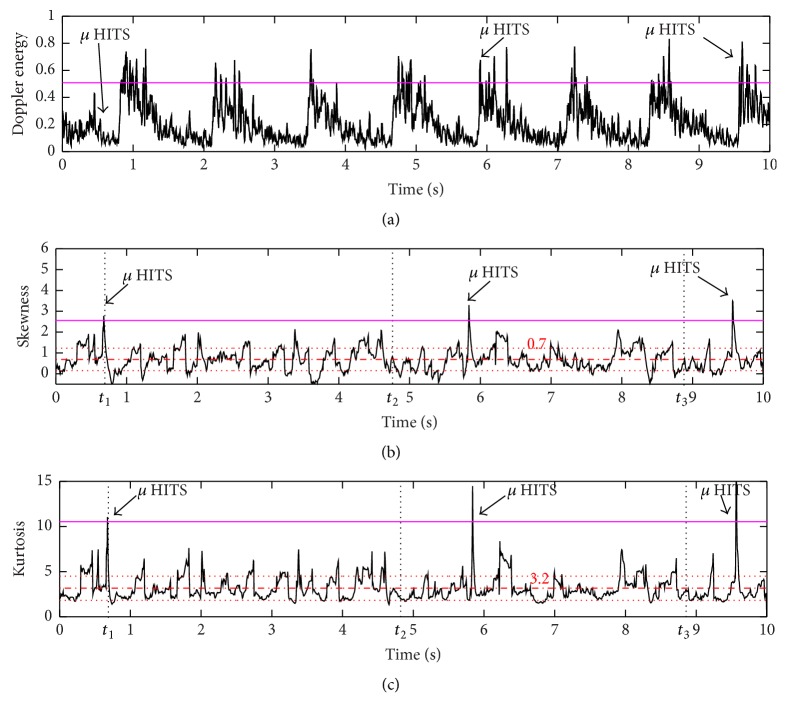
(a) The Doppler energy signal. An empirical threshold is applied to obtain the microembolic standard detection. (b) Skewness signal calculated from the windowed energy signal. A data-based threshold is applied to complete the microembolic detection. The mean value of the skewness signal is 0.7. (c) Kurtosis signal calculated from the windowed energy signal. A data-based threshold is applied to complete the microembolic detection. The mean value of the kurtosis signal is 3.2. Moreover, we choose in (b) and (c) three time positions: *t*_1_ = 0.72 s during which an embolus is present and *t*_2_ = 4.7 s and *t*_3_ = 8.8 s when no embolus is present. We detect, in the case of absence of embolus, *S*(*t*_2_) ≈ *S*(*t*_3_) ≈ 0.7 and *K*(*t*_2_) ≈ *K*(*t*_3_) ≈ 3.2, while in the presence of embolus we detect *S*(*t*_1_) = 2.8 ≠ *S*(*t*_3_) ≈ 0.7 and *K*(*t*_1_) = 11 ≠ *K*(*t*_3_) ≈ 3.2.

The standard detection methods, to which we compare the new proposed methods, are based on a direct detection of the embolic signatures in the energy signal. An empirical threshold is commonly used. This constant threshold can be fixed empirically by the trained user for the entire examination. It is patient-, operator-, and device-dependent. This threshold is set above the maximal background energy of the Doppler signal when no embolus is present [17], that is, the systolic peak. The microembolic standard detection based on a constant threshold is represented in Figure 2(a).

The main limitation of using such method resides in comparing the energy which is time-varying, to a constant threshold. To match between the time-varying trend of the energy and the threshold, two solutions can be proposed: either a time-varying threshold as in [14, 15, 18] that matches the time-varying trend of the decision information or a constant threshold that matches the energy while removing the time-varying trend.

### 2.3. Unit C: Skewness and Kurtosis Based Microembolic Detection

As previously mentioned, it is a threshold-oriented detection. As shown in Figure 2 weak embolic events are impossible to detect with a constant threshold. One way to overcome this issue is to remove the time-varying trend in the instantaneous Doppler energy. To prove that high order statistics such as the skewness and the kurtosis are suitable candidates to overcome this limitation, consider a Doppler signal free of microembolic events and assume that the statistical distribution remains unchanged whatever the time position is even if the mean *μ*_*i*_(*t*) and the variance *σ*_*i*_^2^(*t*) vary with time. Suppose there exists two Gaussian random variables *X*(*t*_2_) = *N*(*μ*_1_(*t*_2_), *σ*_1_(*t*_2_)) and *X*(*t*_3_) = *N*(*μ*_2_(*t*_3_), *σ*_2_(*t*_3_)). It can be shown for the skewness *S* that *S*(*t*_2_) = *S*(*X*(*t*_2_)) = *S*(*X*(*t*_3_)) = 0 and for the kurtosis *K* that *K*(*t*_2_) = *K*(*X*(*t*_2_)) = *K*(*X*(*t*_3_)) = 3. In this example the skewness and the kurtosis are stationary since *S*(*t*) = 0 and *K*(*t*) = 3 for all *t*. This outcome can be verified whatever the distribution form while it remains unchanged over all time values. The only change occurs in the value of the skewness and the kurtosis but not in their stationarity. Consequently, when a microembolic event occurs at a time position *t*_1_, the distribution changes. The direct consequence is *S*(*t*_1_) ≠ *S*(*t*_2_) and *K*(*t*_1_) ≠ *K*(*t*_2_).

Therefore, we can propose a new detector based on calculating the skewness and kurtosis from the energy signal. The calculations are performed using a sliding window *g*(*t*) where the optimal window length and overlap ratio are set during a training phase (see Results section).

The skewness is the third-order standardized moment. When calculated instantaneously (by the sliding window) on the energy it is given by the following equation:

(3) $$\begin{array}{c} S\left(\;t\;\right)=\frac{E{\left[e\left(t\right)-\mu_{e}\left(t\right)\right]}^{3}}{\sigma_{e}{\left(t\right)}^{3}}. \end{array}$$

The kurtosis is the fourth-order standardized moment. When calculated instantaneously on the energy it is given by the following equation:

(4) $$\begin{array}{c} K\left(\;t\;\right)=\frac{E{\left[e\left(t\right)-\mu_{e}\left(t\right)\right]}^{4}}{\sigma_{e}{\left(t\right)}^{4}}, \end{array}$$

where *μ*_*e*_(*t*) and *σ*_*e*_(*t*) are the instantaneous mean and standard deviation of the energy while *E*[] denotes the expected value.

The microembolic detection based on the skewness and kurtosis signals is represented in Figures 2(b) and 2(c).

In order to complete the detection on the skewness and kurtosis signals, a threshold has to be set in order to pick up the peak signals. We decided to establish a data-based threshold for the skewness and kurtosis signals from their respective means *μ*_*s*_ and *μ*_*k*_ and respective standard deviations *σ*_*s*_ and *σ*_*k*_. This threshold is defined as *λ*_*s*_ = *μ*_*s*_ + *mσ*_*s*_ for skewness and *λ*_*k*_ = *μ*_*k*_ + *mσ*_*k*_ for kurtosis, where *m* is a parameter whose value is adjusted using an optimization training phase in a manner that increases the system's sensitivity and specificity (refer to Results section). The thresholds are represented in Figures 2(b) and 2(c).

## 3. The Holter System and the Protocol

**Figure 3:**
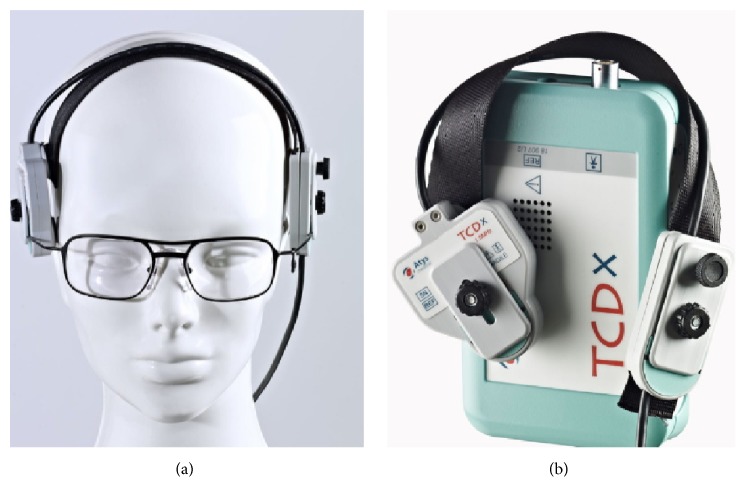
(a) Robot probe and (b) Holter Transcranial Doppler System (TCD-X, Atys Medical, Soucieu en Jarrest, France).

TCD is a noninvasive, nonionizing, inexpensive, portable, and safe technique, which renders it as a convenient tool for the detection of cerebral microemboli. Long time probe positioning and the short effective examination duration are the main limitations of traditional TCD systems. The Transcranial Holter (TCD-X, Atys Medical, Soucieu en Jarrest, France) shown in Figure 3 allows prolonged patient monitoring (higher than 5 hours) with the patient no longer attached to a TCD and does not need to be laying on a bed but rather can be monitored under naturalistic conditions. The Holter is equipped with a robotized automatic probe that helps find the best TCD signal and tracks it automatically during the whole recording.

A database obtained from the Centre Hospitalier Régional Universitaire (CHRU) de Lille (2 Avenue Oscar Lambret, 59000 Lille, France) is used. Informed consent for Holter monitoring was obtained from all monitored patients. The recordings were acquired from the middle cerebral artery of the patients. The ultrasonic wave frequency was 1.5 MHz, the pulse repetition frequency (PRF) was 6.4 kHz, and the ultrasound power was 50 mW/cm^2^.

After the clinical examination, an analogous conversion is performed on the Doppler digital signal and then the Doppler signal is sent to a loudspeaker. From the audible Doppler signal and from the spectrogram displayed on a screen, we detect and count manually the number of microembolic events in order to constitute our gold standard of detection. The gold standard is subject to interagreement between three experts of our laboratory. Then, the positions in time of audibly and visually agreed-on microembolic events are noted. This gold standard is used to assess the results of the different detectors used and validate their performances. Although the gold standard detections obtained from experts and nonexperts might be the same as stated in [19], the experience of the latter experts was useful to distinguish between microembolic signals and artifact signals discussed next. We should also point out that listening to the audio files is made at the normal playing speed and another time at half the normal speed which allows us to detect microemboli previously inaudible due to the well-known temporal and frequency masking effects in audio files.

## 4. Results

The different detectors are tested through algorithms we developed using the numerical calculation software Matlab (Mathworks, Natick, MA, USA). Our database is composed of 18 recorded signals divided into two categories. The first is the training phase (8 signals) dedicated to determining the best settings of the detectors used. The second is the testing phase (10 signals) dedicated to assessing the performances of the detectors used under the optimal settings determined in the training phase.

Two parameters are used to evaluate the detectors:

- Sensitivity (or Detection Rate) calculated as the number of true positive detections/the number of gold standard detections. True positive detection refers to the detection of an embolus recorded in the gold standard.
- Specificity calculated as 1 − False Alarm Rate (FAR) the latter being the number of false positive detections/the total number of detections. False positive detection refers to the detection of an embolus not recorded in the gold standard or in other words an embolus which has not crossed the sample volume.

### 4.1. Training Phase Results

Since the threshold applied on the energy signal to achieve the standard detection is empirically set through the choice of the user, different microembolic detections could be obtained. To overcome this we initialize a training phase to preset the best empirical threshold to be used in the testing phase. 3 to 9 dB values are used.  Table 1 shows the empirical threshold that best maximizes the sensitivity and specificity.

Moreover, since the skewness and kurtosis calculations are performed using a sliding window *g*(*t*) on the energy signal, an experimental test on the training phase signals is initialized to determine the optimal length of the window *g*(*t*) and the optimal overlap ratio. The optimal temporal window length is 7.3 milliseconds and the optimal overlap used is 95%. Also, using these settings we test in the training phase the best data-based threshold *λ*_*s*_ = *μ*_*s*_ + *mσ*_*s*_ and *λ*_*k*_ = *μ*_*k*_ + *mσ*_*k*_ for the skewness and kurtosis signals, respectively. Values of *m* ranging between 3 and 7 are tested.  Table 1 shows the data-based threshold for the skewness and kurtosis signals that best maximizes the sensitivity and specificity.

### 4.2. Testing Phase Results

Table 2 represents the testing phase results for the three different energy detectors. For the standard energy detector with empirical threshold, the sensitivity is 65% and the specificity is 60%. For the energy detector based on skewness calculation the sensitivity is 78% and the specificity is 91%. For the energy detector based on kurtosis calculation the sensitivity is 80% and the specificity is 90%.

The results presented show that the new detectors are able to significantly increase the specificity compared to standard detection (more than 30%). Moreover, the sensitivity achieved by the new detectors is increased by 13% for the skewness detector and 15% for the kurtosis detector compared to that achieved by standard detectors. These results assert the accuracy and superiority of the detection based on skewness and kurtosis calculation of the Doppler energy signal over the standard detection applied directly on the Doppler energy signal.

## 5. Discussion

The results obtained were clear. The methods based on HOS overpassed by far the standard method based on the second-order statistics. The reason explaining such superiority lies in the HOS sensitivity in modifying the distribution form. Knowing that the occurrence of a microembolus superimposed on the Doppler energy signal imposes changes in the distribution of this signal, we propose to use the skewness and kurtosis as new tools for microembolus detection. During embolus-free periods the Doppler energy signals' distribution is fixed and its skewness and kurtosis are never altered. They do not show any variations. However, in the presence of a microembolus superimposed on the energy signal, the skewness and kurtosis signals are altered and the embolus is attributed with a peak whose peakedness level is higher than all the other points of the signal. This detection can outperform standard methods. After being tested on a set of real signals, the skewness and kurtosis based detection offered significant improvements including very high specificity reaching up to 91% and 90%, respectively, compared to 60% achieved by the standard method. In addition, the sensitivity is increased from 65% for standard methods to 78% and 80% for skewness and kurtosis based detectors, respectively.

Consequently, we can affirm that skewness and kurtosis can offer a robust and more reliable detection than standard detection methods and thus can be considered as new techniques for enhancing microembolic detection systems.

In view of the fact that we have proposed 2 detectors, one based on skewness detection and the other on kurtosis detection, it is convenient to give note that the two detectors perform very similarly and yield very close results. The only difference that could be observed is that the kurtosis signal displays small fluctuations around the embolic peak detected while the skewness signal fluctuates more strongly around the embolic peaks. This provides the kurtosis detection with a small advantage in terms of the detection threshold which can be more easily and robustly set.

## 6. Conclusion

In this research study, we propose two detectors based on the calculation of the skewness and kurtosis of the Doppler energy signal, as a tool for an enhanced cerebral microembolus detection. Compared to the standard detector where the detection is performed directly on the energy signal, the skewness and kurtosis based detectors allow increasing of both the sensitivity and the specificity.

This study emphasizes that standard microembolic energy detectors with empirical threshold still pose serious difficulties for the robust detection of microemboli. It also shows that detectors incorporating detection based on skewness and kurtosis calculation from the energy allow a much advanced detection of microemboli, precursors of coming large emboli with strong stroke risks. Thus using these simple and straightforward detectors would be an additional facility boosting the efforts to reduce the occurrence of strokes.

The upcoming step would be attempting to increase the overall performance of the techniques particularly in terms of sensitivity and validating the developed algorithms on a larger database. Moreover, we are on course of including, in the whole detection system, automatic artifact rejection techniques rather than using manual techniques.

## Figures and Tables

**Table 1:** Training phase results of the optimal thresholds that best maximize the sensitivity and specificity for the standard energy detector and skewness and kurtosis based detectors.

	Optimal threshold that maximizes the sensitivity and specificity	Sensitivity (%)	Specificity (%)
Standard energy detector	5 dB	67%	58%
Skewness detector	*λ*_*s*_ = *μ*_*s*_ + 4*σ*_*s*_	76%	91%
Kurtosis detector	*λ*_*k*_ = *μ*_*k*_ + 5*σ*_*k*_	77%	91%

**Table 2:** Results (sensitivity and specificity) for the standard energy detector and the new detectors based on skewness and kurtosis calculations of the Doppler energy signal.

Detector type	True positive	False positive	Sensitivity (%)	Specificity (%)
Gold standard detections = 136				
Standard detection	88	58	65	60
Skewness detection	106	10	78	91
Kurtosis detection	109	12	80	90
